# The feasibility and preliminary effects of a pilot randomized controlled trial: Videoconferencing acceptance and commitment therapy in distressed family caregivers of people with dementia

**DOI:** 10.1177/13591053221141131

**Published:** 2023-01-02

**Authors:** Areum Han, Hon K Yuen, Jeremy Jenkins

**Affiliations:** 1 University of Alabama at Birmingham, USA; 2 Telehealth Private Practice, USA

**Keywords:** anxiety, depression, efficacy, e-health, family, mindfulness, psychological distress, QoL, randomized controlled trial, stress

## Abstract

This pilot randomized controlled trial (RCT) examined preliminary effects of an 8-week videoconferencing acceptance and commitment therapy (ACT) program supplemented with psychoeducation materials on distressed family caregivers of persons living with dementia (PLWD) compared to the control group provided with psychoeducation materials only. Nineteen family caregivers of PLWD in the USA were randomly assigned to the ACT group or the control group. Data was collected at pretest, posttest, and 1-month follow-up (F/U). Compared to the control group, the ACT group showed a significantly larger reduction in grief at posttest, with a medium effect size. Small effects of ACT were found in anxiety, psychological quality of life, and engagement in meaningful activities at posttest and grief, engagement in meaningful activities, and psychological flexibility at F/U compared to the control group. These promising findings warrant a full-scale RCT with adequate power to measure the efficacy of videoconferencing ACT for caregivers of PLWD.

## Introduction

About 16 million people in the USA provide unpaid care for persons living with dementia (PLWD), and the estimated economic value of the unpaid care is about $271 billion (Alzheimer’s Association, 2022). Family caregivers of PLWD report caregiving is stressful with challenges that arise from caregiving situations while often delaying or preventing caregivers from taking care of themselves, which contribute to poorer health and well-being outcomes, such as psychological distress (i.e. symptoms of depression, anxiety, and stress) and decreased participation in personally valued activities (Karg et al., 2018; Ma et al., 2018; Solway et al., 2017). A recent mixed-method systematic review identified these caregivers’ needs in learning how to manage and cope with their own emotions and balancing care demands with other important aspects of life (Bressan et al., 2020).

A meta-analysis study found that the aggregate prevalence of depression and anxiety in family caregivers of PLWD were 34% and 43.6%, respectively (Sallim et al., 2015). These caregivers may feel guilt, frustration, or anger at themselves when they have negative thoughts and emotions arising from challenging caregiving situations and try to control or avoid these thoughts and emotions (Chan et al., 2010; Gallego-Alberto et al., 2021). For example, these caregivers may feel guilty in having anger while caring for PLWD and feeling that they are not doing enough or doing wrong, which may contribute to psychological distress (Feast et al., 2017; Gallego-Alberto et al., 2021; Han et al., 2022). Caregivers also may feel sad when thinking about the lost abilities of their relatives and have excessive worries or fears about the uncertain future with the progression of dementia (Tatangelo et al., 2018). In addition, family caregivers of PLWD may feel they lack time and energy to participate in other valued activities while focusing on caregiving roles (Tatangelo et al., 2018).

Family caregivers often experience loss and grief while caring for PLWD, with a prevalence ranging from 47% to 71% (Chan et al., 2013). Studies suggest that loss and grief in caregivers of PLWD should be acknowledged and addressed to prevent caregivers from struggling to fight the inevitable decline in PLWD, which can contribute to caregivers’ feelings of helplessness, perceived caregiving burden, and depressive symptoms and to nursing home admission in PLWD (Liew et al., 2019; Liew and Yap, 2020; Walker and Pomeroy, 1997). For example, longitudinal cohort studies found the direct effects of grief on predicting depression in family caregivers of PLWD at 2.5 years and poorer quality of life at 6 months (Liew et al., 2019, 2020).

Investigators have made great efforts to develop and test interventions for family caregivers of PLWD, especially to meet care-related needs, but relatively fewer studies have investigated interventions focusing on improving these caregivers’ mental health and participation in their valued activities (Kishita et al., 2018). For example, meta-analyses found no significant effect of skill-building psychoeducation interventions on these caregivers’ mental health outcomes, such as depressive symptoms (Kishita et al., 2018). Limited evidence exists, having a small effect of skill-building psychoeducation interventions on caregiving burden and small effects of cognitive behavioral therapy (CBT) on caregivers’ depressive symptoms and anxiety (Kishita et al., 2018).

A growing body of literature suggests mindfulness-based interventions show promise for improving mental health outcomes in family caregivers of PLWD, including depressive symptoms, anxiety, stress, and QoL (Han, 2022). Mindfulness is defined as “awareness that arises through paying attention, on purpose, in the present moment, and nonjudgmentally” (Kabat-Zinn, 1994: 4). Studies also have found that mindfulness was negatively related to depressive symptoms, anxiety, stress, and grief in family caregivers of PLWD (Jain et al., 2019; Murfield et al., 2020). However, there is limited evidence for mindfulness-based interventions in family caregivers of PLWD, and it is not known which type and delivery mode of mindfulness-based interventions are effective for improving these caregivers’ mental health outcomes (Collins and Kishita, 2019; Han, 2022; Kishita et al., 2018).

Acceptance and commitment therapy (ACT) is an emerging evidence-based approach that can promote mental health and engagement in personally valued activities through acceptance and mindfulness processes and behavior change processes for valued living (Hayes et al., 2012a). ACT is based on a psychological flexibility model involving these six psychological processes that serve as mechanisms of change: (a) acceptance (i.e. being open to unwanted thoughts and emotions as they are); (b) cognitive defusion (i.e. stepping back or detaching from unhelpful thoughts and emotions to reduce their dominance over behaviors); (c) being present (i.e. maintaining voluntary and flexible contact with the present moment); (d) observing self (i.e. flexible self-conceptualization and perspective taking); (e) values (i.e. identifying and connecting values to behaviors for a meaningful life); and (f) committed action (i.e. establishing patterns of behaviors to live a meaningful life aligned with values; Hayes et al., 2012a). Two recent meta-analysis studies found small to medium effects of ACT on depressive symptoms, anxiety, stress, QoL, and psychological flexibility in family caregivers of people with chronic conditions (Han et al., 2020, 2021). Previous ACT studies also found promising results that showed effects of ACT on reducing caregivers’ grief, guilt, perceived caregiving burden, and cognitive fusion and promoting self-compassion and engagement in meaningful or valued activities (Davis et al., 2020; Han et al., 2020, 2022). Psychological inflexibility (i.e. the opposite of psychological flexibility) was shown to be a key factor significantly associated with depression and anxiety in family caregivers of PLWD (Kishita et al., 2020).

To our knowledge, there have been eight studies so far that implemented ACT for family caregivers of PLWD. Two full-scale randomized controlled trials (RCTs) conducted in Spain for depressed family caregivers of PLWD found statistically significant effects of eight weekly individual in-person ACT sessions on decreasing depressive symptoms and anxiety compared to the control condition with a 2-hour psychoeducation session on dementia (Losada et al., 2015; Márquez-González et al., 2020). Two pilot studies conducted in Spain involved a group-based in-person guilt-focused intervention based on CBT, ACT, and compassion-focused therapy approaches and found promising findings, such as significant reductions in depression, anxiety, and guilt over time (Gallego-Alberto et al., 2021; Romero-Moreno et al., 2022). ACT delivered online may be an efficacious and acceptable alternative to in-person services that can improve care accessibility and flexibility in scheduling for caregivers, who often cannot leave their relatives with dementia alone (Thompson et al., 2021).

Four small-scale one-group pretest-posttest design studies were conducted in the USA (three studies) and in the UK (one study) that involved six weekly individual telephone-based ACT sessions (Fowler et al., 2021), ten self-guided online ACT sessions plus online dementia education resources (Fauth et al., 2022), eight guided self-help online ACT sessions and access to an online peer support group (Kishita et al., 2022), and ten weekly coach-guided individual videoconferencing ACT sessions plus psychoeducation materials (Han et al., 2022).

These studies found promising results, including decreased depressive symptoms, anxiety, stress, and caregiving burden after participants completed ACT sessions, and evidence to support the feasibility and acceptability of ACT sessions delivered by methods other than in-person sessions (i.e. phone-based therapy, self-guided online modules, and videoconferencing sessions; Fauth et al., 2022; Fowler et al., 2021; Han et al., 2022; Kishita et al., 2022). However, none of these studies were RCTs with a control group and random assignment, a design regarded as the gold standard, as RCTs are less susceptible to biases than studies with other designs for assessing therapeutic interventions (Bondemark and Ruf, 2015). The feasibility of participant recruitment and retention in RCTs can differ from that in one-group pretest-posttest design studies due to additional psychological burdens in participants that arise from the RCT process, such as a lack of control and anxiety that result from random allocation and disappointment when allocated to a less favorable control group condition (Naidoo et al., 2020).

Because there are a few current studies involving ACT for family caregivers of PLWD, conducting a small-scale RCT before initiating a full-scale RCT will be helpful, especially to test the feasibility of participant recruitment and retention in studies involving a control group condition and random allocation to groups and the preliminary effects of videoconferencing ACT on these caregivers compared to the control group condition. The current study, therefore, aimed to test the feasibility of participant recruitment and retention in a pilot RCT in which distressed caregivers of PLWD were randomly assigned to either an 8-week, coach-guided, videoconferencing, individual ACT group provided with psychoeducation materials or to a control group provided with psychoeducation materials only. This study also aimed to test the preliminary effects of ACT on psychological distress (i.e. depressive symptoms, anxiety, and stress), perceived caregiving burden, grief, guilt, psychological QoL, and measures relevant to ACT processes (i.e. psychological flexibility, cognitive fusion, engagement in meaningful activities, and self-compassion) in distressed caregivers of PLWD compared to the control group. To our knowledge, this pilot study is the first RCT to investigate ACT for family caregivers of PLWD in the USA and to involve online ACT for these caregivers in the world.

## Methods

### Design and participants

This pilot project employed a two-arm parallel-group RCT design. Participants were recruited based on the following eligibility criteria. Inclusion criteria involved: (1) community-dwelling adults (aged 18 years or older) who were taking primary responsibility for the care of a relative with dementia; (2) having at least mild symptoms of psychological distress (scores ⩾5 in the Depression Subscale, scores ⩾4 in the Anxiety Subscale, or scores ⩾8 in the Stress Subscale) as measured by the Depression, Anxiety, and Stress Scale (DASS-21; Antony et al., 1998); and (3) having a computer or a smartphone with internet access at home. Exclusion criteria involved: (1) having cognitive, physical, or sensory deficits or language barriers (non-English communicator) that might impede study participation; (2) receiving a psychological therapy at the time of recruitment; (3) having a prior experience in ACT; (4) having psychiatric hospitalizations in the previous 2 years; (5) taking medications for managing severe mental illness (such as psychosis) or seizure at the time of recruitment; (6) considering or planning to place relatives with dementia in a nursing home within 4 months; or (7) recent frequent hospitalizations of their relatives with dementia that might contribute to study dropout. The study’s sample size was derived from a method for estimating sample sizes for pilot trials. For a pilot trial designed with 80% power at a significance level of 0.05, the method recommends at least 10 participants per group as the pilot trial sample size for an intervention (Whitehead et al., 2016).

### Procedures

A study invitation letter with the project coordinator’s contact information was shared via email with directors of Area Agencies on Aging and community-based programs for people with dementia and their caregivers (e.g. adult day care programs) in Alabama (primary) and a few other USA states. When interested potential participants contacted the project coordinator, the coordinator went through the eligibility screening and informed consent process. Hard copy written consent forms and questionnaires were mailed to potential participants with a pre-addressed pre-stamped envelope for their return enclosed in the mailing. Following pretest evaluation, participants were randomly assigned to either the ACT group or the control group with a 1:1 allocation ratio. The co-investigator (Dr. Yuen), who had no contact with participants, generated the randomization sequence using a computer random number generator and handed the project coordinator the sequentially numbered opaque sealed envelopes containing the participant group allocation list. When the project coordinator received the signed consent form and completed pretest questionnaire from participants, she opened the envelope that was matched to the participant’s study ID number and informed them of their assigned group. A graduate student research assistant, who was blinded to the group allocation, was responsible for data entry and management. All participants completed questionnaires again 8 and 12 weeks after completing their pretest evaluations.

The study was conducted with Institutional Review Board (IRB) approval from the University of Alabama at Birmingham (IRB-300007215). The trial was registered at ClinicalTrials.gov (NCT04847986) before enrollment of the first participant, and the study was conducted from mid-July 2021 to mid-June 2022.

### The ACT and control groups

Participants in the ACT group received individual ACT sessions guided by a trained ACT coach (Mr. Jenkins) through Zoom videoconferencing 1 hour per week for 8 weeks. The overview of the eight weekly ACT sessions are reported in Supplemental Table 1. The ACT protocol for distressed family caregivers of PLWD was developed considering ACT resources, including ACT protocols for other populations (e.g. veterans with depression), ACT metaphors and exercises, and a book chapter addressing the use of ACT in family caregivers of PLWD (e.g. Ahles and Jenkins, 2017; Hayes et al., 2012b; Márquez-González et al., 2010; Walser et al., 2012). The ACT protocol was structured around the six processes of ACT strengthened by behavioral activation (BA) techniques (e.g. activity scheduling and monitoring) to add a structured format for action plans and to maximize benefits for mental health outcomes (Han and Kim, 2022a). The protocol was then reviewed by two ACT experts with prior experience in developing the ACT protocol and with the clinical expertise and experiences in ACT to revise the protocol as needed. In addition, our previous preliminary one-group pretest-posttest design study tested the feasibility and acceptability of the protocol for distressed family caregivers of PLWD (Han et al., 2022). We strengthened the protocol by adding one additional in-session worksheet to address grief in particular based on a participant’s suggestion to have more time to address grief in our preliminary study and more ACT resources for between-session practice after our preliminary study was conducted.

Hard copies of ACT session worksheets were mailed to the participants in the intervention group before the first ACT session. The ACT coach, a licensed professional counselor with substantial clinical experiences and expertise in ACT, delivered ACT sessions to all participants in the intervention group. Active interactions with the coach and engagement with the materials were encouraged using various in-session learning activities (e.g. in-session worksheets, metaphors, and case scenario exercise) and homework assignment. For example, the coach and the participant worked on learning activities, which helped the participant acknowledge that it was normal to have negative emotions and thoughts in stressful care situations and accept those emotions and thoughts, such as feelings of guilt arising from a mistake or a thought that he or she was not doing enough to be a perfect caregiver. After each session, the coach sent each participant additional online ACT resources matched to weekly learning aims (i.e. guided ACT exercise video clips) and a summary of strategies and skills covered during the session to encourage participants to practice between sessions. Participants were also provided with links to short video clips and hard copies of psychoeducation materials as supplemental resources before the first ACT session to better meet the needs of this population. These psychoeducation resources were aimed at increasing caregivers’ understanding of dementia (e.g. types, stages, and symptoms); care strategies for different stages of dementia; BPSD in PLWD and how to manage these behaviors; and communication strategies. Participants in the control group maintained care as usual and received the same psychoeducation materials given to the participants in the ACT group during the study period. After the study period ended, the control group received links to online ACT resources.

### Feasibility outcomes

The feasibility of recruitment, eligibility, and retention of participants was evaluated by tracking the number of potential participants who contacted the study team and went through the eligibility screening, the number of those excluded with reasons for exclusion, the number of participants who consented, the number of dropouts, and the duration to recruit the target sample size. Adherence to the intervention by participants was determined through the session attendance rates in the ACT group. The coach completed the session logs, and the principal investigator who developed the protocol (Dr. Han) reviewed the logs to make sure the coach adhered to the protocol across participants.

### Data collection

Quantitative data generated by self-reported questionnaires was collected at pretest (i.e. baseline), posttest (i.e. 8 weeks after pretest questionnaire completion), and 1-month follow-up (F/U; i.e. 4 weeks after posttest questionnaire completion) for both groups.

The DASS-21 is a 21-item questionnaire assessing depressive symptoms, anxiety, and stress over the past 7 days on a scale of 0–3 (Antony et al., 1998). Higher scores indicate greater symptomatology in each subscale with cutoff scores (Antony et al., 1998). The DASS-21 has acceptable to excellent internal consistency and concurrent validity in clinical and non-clinical populations (Antony et al., 1998; Henry and Crawford, 2005).

The Short version of the Zarit Burden Interview (ZBI) is a 12-item self-report questionnaire assessing caregivers’ perceived burden on a scale of 0–4 (Bédard et al., 2001). Higher scores indicate higher levels of perceived caregiving burden. The ZBI has good internal consistency and convergent and discriminant validity in caregivers of PLWD (Bédard et al., 2001; Pinyopornpanish et al., 2020).

The Marwit–Meuser Caregiver Grief Inventory—Brief-Form (MM-CGI-BF) is a six-item self-report questionnaire assessing grief in caregivers of PLWD (i.e. losses, including anticipation of future losses) on a scale of 1–5 (Liew et al., 2020). Higher scores indicate higher levels of grief. The MM-CGI-BF has good internal consistency, test-retest reliability, and construct validity in caregivers of PLWD (Liew et al., 2020).

The Caregiver Guilt Questionnaire (CGQ) is a 22-item self-report questionnaire assessing feelings of guilt in caregivers on a scale of 0–4 (Losada et al., 2010). Higher scores indicate higher levels of guilt. The CGQ has good internal consistency and convergent validity in caregivers of PLWD (Losada et al., 2010).

The World Health Organization Quality of Life-BREF (WHOQOL-BREF)—Psychological quality of life (QoL) component (Skevington et al., 2004) has six items measuring psychological quality of life over the last 2 weeks on a scale of 1–5. Higher scores denote higher psychological QoL. The entire measure and its psychological QoL component have good to excellent internal consistency, discriminant validity, and construct validity (Skevington et al., 2004).

The Acceptance and Action Questionnaire-II (AAQ-II) is a 7-item self-report questionnaire measuring psychological flexibility on a scale of 1–7 (Bond et al., 2011). Higher scores indicate poor psychological flexibility. The AAQ-II has good internal consistency, test-retest reliability, concurrent validity, convergent validity, and divergent validity (Bond et al., 2011; Eisenbeck et al., 2018).

The Cognitive Fusion Questionnaire (CFQ)-7 is a seven-item self-report questionnaire measuring cognitive fusion on a scale of 1–7 (Gillanders et al., 2014). Higher scores indicate a higher degree of cognitive fusion. The CFQ-7 has good to excellent internal consistency, test-retest reliability, concurrent validity, and divergent validity (Gillanders et al., 2014).

The Engagement in Meaningful Activities Survey (EMAS) is a 12-item self-report questionnaire assessing a person’s subjective experience of the meaningfulness of everyday activities on a scale of 1–4 (Goldberg et al., 2002). Higher scores indicate greater levels of engagement in meaningful activities. The EMAS has good internal consistency, test-retest reliability, and convergent validity (Eakman et al., 2010).

The Self-Compassion Scale-Short Form (SCS-SF) has 12 items assessing the relationship of self-compassion to positive psychological health on a scale of 1–5 (Raes et al., 2011). Higher scores indicate higher levels of self-compassion. The SCS-SF has good internal consistency, test-retest reliability, and convergent validity in clinical populations (Hayes et al., 2016).

### Data analysis

Composite scores with appropriate reverse coding of the responses in the outcome measures were computed for analysis. Descriptive statistics were used to summarize participants’ characteristics, and the Shapiro–Wilk test was used to test the normality of data. The chi-squared test (χ^2^ test)/Fisher’s exact test (for categorical variables), Mann–Whitney *U* test (for continuous variables; data with non-normal distribution), or independent *t*-tests (for continuous variables; data with normal distribution) were used to test the equivalence of the groups in general characteristics and outcome measures at pretest.

We compared the change scores of the outcome measures at posttest and at 1-month F/U from pretest between the ACT and control groups and used nonparametric statistical tests because these change scores did not meet the assumptions of normality. Specifically, the Mann–Whitney *U* test was used to evaluate significant differences in the median values of the change scores in the outcome measures between the intervention and control groups. The Wilcoxon signed rank test was used to test significant differences in the median values of the change scores in the outcome measures across the study time points within each of the groups.

We used intention-to-treat analysis (i.e. data from all participants are analyzed as randomized); one participant in the control group, who did not complete the posttest and the 1-month F/U evaluation, was included for analysis. The last observation carried forward strategy was used to impute these missing values by considering that this participant did not have any changes in outcome measures at each subsequent evaluation. Statistical significance was set at *p* ⩽ 0.05, two-sided. Because of the exploratory nature of the study, no adjustment of *p* values was conducted for multiple statistical comparisons of the outcome measures (Rothman, 1990). Effect size was calculated by dividing the z statistic by the square root of the total number of observations and then taking the absolute value as the effect size (Fritz et al., 2012). All data analyses were conducted using the IBM SPSS Statistics 28.0.

## Results

### Participant flow and feasibility outcomes

Supplemental Figure 1 shows the flow of participants. Forty-three potential participants contacted the research team and were screened for study eligibility over the 7-month recruitment period. Of these, eighteen did not meet the eligibility criteria because of the following reasons: nine participants did not meet DASS-21 screening criteria; four participants were receiving a psychological therapy; three participants were receiving a psychological therapy and had a prior experience in ACT; one participant had a prior experience in ACT; and one participant had frequent hospitalizations of the PLWD that might have caused study dropouts and impact the caregiver’s mental health outcomes. Six participants, who were eligible for study participation, chose not to participate in the study and did not give consent (unknown reasons). The remaining 19 participants gave informed consent, completed pretest questionnaires, and were randomly assigned to the ACT group or the control group. All participants except one completed posttest and 1-month F/U evaluations. One participant from the control group was lost after completing the pretest evaluation and group assignment (no response). All participants assigned to the ACT group completed all eight ACT sessions. The coach adhered to the protocol across participants.

### Baseline characteristics of the participants

Supplemental Table 2 shows characteristics of all participants (*N* = 19) with characteristics of each group and results of between-group differences in baseline characteristics. There was no statistically significant difference in characteristics (Supplemental Table 2) and outcome measures at pretest (Supplemental Table 3) between the ACT and control groups (Supplemental Material). All participants were females, and the majority were daughters of PLWD (78.9%). The age of participants ranged from 18 to 78 years (mean: 54.6 years). The majority of participants were either African American (47.4%) or non-Hispanic White (42.1%). Years since the relative’s diagnosis of dementia ranged from 1 to 10 years (mean: 4 years), and the majority of the participants’ relatives (about 58%) were in the middle stage of dementia.

### Effects of the videoconferencing ACT program on family caregivers of PLWD

Supplemental Tables 4 and 5 report scores in outcome measures at pretest, posttest, and 1-month F/U for each group (Supplemental Table 4), change scores at posttest and 1-month F/U from the pretest of each group (Supplemental Table 5), and findings of within-group comparisons (Supplemental Table 4) and between-group comparisons (Supplemental Table 5).

Compared to the control group, the ACT group showed a statistically significantly larger reduction in grief from pretest to posttest (*p* = 0.035), with an effect size (*r* = 0.49) close to medium (*r* = 0.5; Cohen, 1988), but not from pretest to 1-month F/U (*p* = 0.243; Supplemental Table 5). No statistically significant difference in change scores of the other outcome measures between the ACT and control groups was observed. However, small effects of the ACT program were found in anxiety (*r* = 0.23), psychological QoL (*r* = 0.32), and engagement in meaningful activities (*r* = 0.33) at posttest and in grief (*r* = 0.28), engagement in meaningful activities (*r* = 0.22), and psychological flexibility (*r* = 0.44) at 1-month F/U compared to the control group.

For within-group comparisons in the ACT group, there were statistically significant reductions in stress at posttest (*p* = 0.011), grief at posttest (*p* = 0.049) and 1-month F/U (*p* = 0.049), guilt at posttest (*p* = 0.044), and psychological inflexibility at 1-month F/U (i.e. improvements in psychological flexibility; *p* = 0.034) and improvements in psychological QoL at posttest (*p* = 0.05) and 1-month F/U (*p* = 0.35) and self-compassion at 1-month F/U (*p* = 0.049) compared to the pretest (Supplemental Table 4). Medium effects of ACT were found in stress at posttest (*r* = 0.60), psychological QoL at 1-month F/U (*r* = 0.50), and psychological flexibility at 1-month F/U (*r* = 0.50) compared to the pretest. Compared to the pretest, small effects of ACT were found in the following outcomes: depressive symptoms at posttest (*r* = 0.27); anxiety at posttest (*r* = 0.31); stress at 1-month F/U (*r* = 0.35); perceived caregiving burden at posttest (*r* = 0.28) and 1-month F/U (*r* = 0.38); grief at posttest (*r* = 0.46) and 1-month F/U (*r* = 0.46); guilt at posttest (*r* = 0.48) and 1-month F/U (*r* = 0.28); psychological QoL at posttest (*r* = 0.46); psychological flexibility at posttest (*r* = 0.32); engagement in meaningful activities at posttest (*r* = 0.43) and 1-month F/U (*r* = 0.43); and self-compassion at posttest (*r* = 0.42) and 1-month F/U (*r* = 0.46).

## Discussion

As a small-scale pilot RCT, the present study was not adequately powered, but found promising results. Compared to the control group provided with psychoeducation materials only, the ACT group supplemented with psychoeducation materials showed a significantly larger reduction in grief from pretest to posttest, with a medium effect size. Such statistically significant reductions in grief in the ACT group compared to the control group are due to the ACT group’s statistically significant reduction in grief at posttest compared to the pretest (*p* = 0.049 in Supplemental Table 4; the mean and median changes in Supplemental Table 5) while the control group showed an increase in grief at posttest compared to pretest (not statistically significant in Supplemental Table 4; the mean and median changes in Supplemental Table 5).

Studies found grief in family caregivers was greatest when their relatives had moderate to severe stages of dementia, and that their grief can increase over time due to gradual deterioration of dementia and gradual loss of selfhood in PLWD (Chan et al., 2013; Cheung et al., 2018). About 80% of participants in the present study reported their relatives’ stage of dementia as moderate to severe at pretest (77.8% in the ACT group and 80% in the control group), and the mean pretest scores in grief in both ACT and control groups were quite high, which was higher than those found in participants in previous cohort studies with large sample sizes (Liew et al., 2019, 2020). Psychoeducation materials provided to the control group in the present study did not have a specific intervention component to address caregivers’ grief, but there was a specific intervention component aligned with the ACT model during the ACT sessions to acknowledge and address grief and loss.

In addition, grief was measured using the MM-CGI-BF, which was specifically developed to assess grief in caregivers of PLWD and showed good reliability and validity (Liew et al., 2020). The sensitivity and specificity of 0.90 at the optimal cutoff score of more than or equal to 21 was found in a previous psychometric study, which suggests the MM-CGI-BF is a useful assessment tool for identifying caregivers with high levels of grief who are likely to benefit from clinical trials addressing grief (Liew et al., 2020). Although the level of grief at pretest was higher in the ACT group compared to the control group, there was no statistically significant difference between groups at pretest.

In summary, findings of grief in the present study can be explained by the participants’ characteristics (i.e. high levels of grief in both groups as caregivers of relatives of people with moderate to severe stages of dementia), the intervention component (i.e. ACT addressing grief vs. psychoeducation not particularly addressing grief), and the use of a reliable valid grief measure specifically developed for caregivers of PLWD (the MM-CGI-BF). According to recent systematic reviews regarding ACT for family caregivers of people with chronic conditions (Han et al., 2020, 2021), only one study (Davis et al., 2020) considered grief as an outcome measure. Davis et al. (2020) found no statistically significant difference in grief between the self-help ACT group supported with phone calls and the usual care control group in caregivers of patients receiving palliative care, which might be due to the small sample size. However, the study showed a small effect of ACT on reducing grief compared to the control group. Although findings in grief in the present study should be confirmed by a full-scale RCT, future studies may consider addressing grief in particular within the ACT protocol for family caregivers of PLWD and measuring effects of ACT on grief as an outcome.

The present study found small effects of ACT in anxiety at posttest, grief at 1-month F/U, psychological QoL at posttest, psychological flexibility at 1-month F/U, and engagement in meaningful activities at posttest and 1-month F/U compared to the control group, but statistically significant differences between groups were not found in these outcomes, possibly due to the small sample size for group comparisons. These findings are supported to some extent by findings of previous full-scale RCTs in Spain, which found medium effects of eight weekly individual in-person ACT sessions on decreasing anxiety and increasing participation in leisure activities at posttest compared to control groups provided with a 2-hour psychoeducation session on dementia (Losada et al., 2015; Márquez-González et al., 2020). Also, the findings are supported by recent meta-analysis studies in family caregivers of people with chronic conditions, which found small effects of ACT on anxiety and QoL at posttest and small to medium effects of ACT on psychological flexibility at posttest and follow-ups compared to control groups (Han et al., 2020, 2021).

In particular, there was no statistically significant between-group difference in depressive symptoms at posttest and 1-month F/U compared to the pretest. These results could be because the present small-scale RCT was not powered enough to show between-group differences, and the study involved family caregivers who had at least mild symptoms of psychological distress (i.e. at least mild depressive symptoms, anxiety, or stress) rather than focusing on depressed caregivers. When participants with at least mild depressive symptoms were included for the additional secondary analyses only (*N* = 12; six per group), results showed more positive findings even with this smaller sample size, including small to medium effects of ACT on decreasing depressive symptoms (*r* = 0.37), anxiety (*r* = 0.40), stress (*r* = 0.42), grief (*r* = 0.63), and psychological inflexibility (*r* = 0.51) and increasing engagement in meaningful activities (*r* = 0.72) compared to the control group. A recent meta-analysis found a statistically significant larger effect of internet-based ACT on decreasing depressive symptoms when studies targeted people with depressive symptoms in particular (Han and Kim, 2022b). These findings suggest ACT may be most effective in varied mental health outcomes when caregivers are screened to target the subpopulation with depressive symptoms in particular.

For the within-group comparisons across time, the ACT group showed statistically significant reductions in stress, grief, guilt, and psychological inflexibility and improvements in QoL and self-compassion, which are supported to some extent by previous ACT studies for caregivers of PLWD (Davis et al., 2020; Han et al., 2020, 2022). The present study also found small to medium effects of ACT at posttest and/or 1-month F/U in almost all outcomes compared to pretest, which are also supported by previous studies for caregivers, including family caregivers of PLWD (e.g. Han et al., 2020, 2021, 2022). These promising findings may indicate the possibility of showing the efficacy of ACT for this population in a full-scale RCT with adequate power to detect the intervention effects.

The control group provided with psychoeducation materials showed statistically significant reductions in stress and guilt and improvements in self-compassion at posttest and 1-month F/U compared to pretest, which indicates benefits from the evidence-based psychoeducation materials (Supplemental Table 4). The evidence-based psychoeducation materials involved specific contents regarding care-related strategies (e.g. how to manage BPSD), emotions caregivers and PLWD may experience, and tips to take care of themselves. These participants might show reduced stress by learning specific care strategies from these psychoeducation materials and reduced guilt and improved self-compassion by understanding that it is natural and okay to have certain emotions and thoughts as caregivers and they have to take care of themselves. More than half of the participants in the present study had never attended support groups, through which caregivers typically receive information, learn caregiving strategies from care professionals, and share their experiences with other caregivers. Such care-related characteristics may explain why the control group provided with psychoeducation materials only showed these benefits.

Several limitations should be considered when interpreting the findings of the present study. Because it was a small-scale pilot RCT, its findings should be confirmed by a future full-scale RCT with adequate power to detect the efficacy of ACT. The present study involved a convenience sampling method, and all participants were female. About 67% of primary family caregivers of PLWD in the USA are female (Alzheimer’s Association, 2022), and female caregivers of PLWD are 1.5 times more likely to have depression and 2.5 times more likely to have anxiety than male caregivers (Sallim et al., 2015), so we anticipated that this small-scale study with a convenience sampling approach might involve female caregivers alone. About 67% of family caregivers of PLWD in the USA are non-Hispanic White, and 10% are African American (Alzheimer’s Association, 2022). About 47% of the participants in the present study were Black or African American because most of its participants lived in Alabama, where the proportion of individuals who are Black or African American is higher than that of most other states. A future full-scale RCT may consider conducting a multi-site study to ensure recruitment of diverse ethnic groups and to test if there are differential effects of ACT on caregivers mediated by caregivers’ race or ethnicity.

Although the present study did not screen caregivers who were experiencing high levels of grief, the participants’ pretest scores in grief were quite high, reflecting the high prevalence of experiencing grief while caring for PLWD (Chan et al., 2013). Further studies may directly target caregivers who experience grief but have not clinically significant depression yet to use ACT to prevent depression for this population as studies found the direct effects of grief on predicting depression at 2.5 years (Liew et al., 2019). The present study involved a relatively shorter time for F/U evaluation due to the research funding period. A full-scale RCT with a longer time for F/U evaluations should be conducted to assess the maintenance of treatment effects.

This pilot study was the first RCT that investigated ACT for family caregivers of PLWD in the USA and that involved videoconferencing ACT for these caregivers in the world. Forty-three potential participants contacted the research team with interest in the study participation over the 7-month recruitment period. This number is promising considering that our research team did not have the access to the population through clinics, and recruitment relied on sharing our study invitation letter in the community by e-mail only. All participants in the ACT group completed all eight ACT sessions, and only one person in the control group dropped from the study after the group assignment. Although this was a small scale study that was not adequately powered, findings were promising, including a significantly larger reduction in grief at posttest in the ACT group with a medium effect size, compared to the control group. Future full-scale RCTs are needed to assess the efficacy of ACT for family caregivers of PLWD.

## Supplemental Material

sj-doc-1-hpq-10.1177_13591053221141131 – Supplemental material for The feasibility and preliminary effects of a pilot randomized controlled trial: Videoconferencing acceptance and commitment therapy in distressed family caregivers of people with dementia Click here for additional data file. Supplemental material, sj-doc-1-hpq-10.1177_13591053221141131 for The feasibility and preliminary effects of a pilot randomized controlled trial: Videoconferencing acceptance and commitment therapy in distressed family caregivers of people with dementia by Areum Han, Hon K Yuen and Jeremy Jenkins in Journal of Health Psychology

sj-doc-2-hpq-10.1177_13591053221141131 – Supplemental material for The feasibility and preliminary effects of a pilot randomized controlled trial: Videoconferencing acceptance and commitment therapy in distressed family caregivers of people with dementia Click here for additional data file. Supplemental material, sj-doc-2-hpq-10.1177_13591053221141131 for The feasibility and preliminary effects of a pilot randomized controlled trial: Videoconferencing acceptance and commitment therapy in distressed family caregivers of people with dementia by Areum Han, Hon K Yuen and Jeremy Jenkins in Journal of Health Psychology

sj-docx-3-hpq-10.1177_13591053221141131 – Supplemental material for The feasibility and preliminary effects of a pilot randomized controlled trial: Videoconferencing acceptance and commitment therapy in distressed family caregivers of people with dementia Click here for additional data file. Supplemental material, sj-docx-3-hpq-10.1177_13591053221141131 for The feasibility and preliminary effects of a pilot randomized controlled trial: Videoconferencing acceptance and commitment therapy in distressed family caregivers of people with dementia by Areum Han, Hon K Yuen and Jeremy Jenkins in Journal of Health Psychology
