# Supplementary material for: The feasibility and preliminary effects of a pilot randomized controlled trial: Videoconferencing acceptance and commitment therapy in distressed family caregivers of people with dementia
Source: J Health Psychol. 2023 Jan 2;28(6):554–67. doi: 10.1177/13591053221141131 (PMC10119897; doi:10.1177/13591053221141131)
Supplement: sj-doc-2-hpq-10.1177_13591053221141131 – Supplemental material for The feasibility and preliminary effects of a pilot randomized controlled trial: Videoconferencing acceptance and commitment therapy in distressed family caregivers of people with dementia [file sj-doc-2-hpq-10.1177_13591053221141131.doc]

**Enrollment**

Assessed for eligibility (*n* = 43)

Participated in the posttest evaluation (*n* = 9)

Allocated to the control group (*n* = 10)

Excluded (*n* = 24)

- Did not meet eligibility criteria (*n* = 18)

- Declined to participate (*n* = 6)

Participated in the pretest evaluation and randomized (*n* = 19)

Allocated to the intervention group (*n* = 9)

**Allocation**

**Posttest**

Participated in the posttest evaluation (*n* = 9)

**Follow-up & analysis**

Participated in the 1-month

- follow-up evaluation (*n* = 9)

Included for analysis (*n* = 9)

Participated in the 1-month

- follow-up evaluation (*n* = 9)

Included for analysis (*n* = 10)

**Figure S1.** Participant flow diagram.
