# Supplementary material for: The feasibility and preliminary effects of a pilot randomized controlled trial: Videoconferencing acceptance and commitment therapy in distressed family caregivers of people with dementia
Source: J Health Psychol. 2023 Jan 2;28(6):554–67. doi: 10.1177/13591053221141131 (PMC10119897; doi:10.1177/13591053221141131)
Supplement: sj-docx-3-hpq-10.1177_13591053221141131 – Supplemental material for The feasibility and preliminary effects of a pilot randomized controlled trial: Videoconferencing acceptance and commitment therapy in distressed family caregivers of people with dementia [file sj-docx-3-hpq-10.1177_13591053221141131.docx]

**Supplementary Materials**

**Table S1. Overview of the 8 weekly acceptance and commitment therapy (ACT) sessions**

| Sessions | Aims |
| --- | --- |
| Week 1. Orientation and identifying difficulties in ACT processes | - Orienting the caregiver to the intervention sessions  - Conducting an initial interview to identify the caregiver’s difficulties in six ACT processes  - Introducing basic concepts of ACT using metaphors  ***Learning materials***: ACT initial case conceptualization form & ACT in a nutshell metaphor |
| Week 2. ACT (Creative Hopelessness) | - Helping the caregiver explore the efforts he or she has made to try to eliminate, change, or fix his or her negative internal experiences (i.e., difficult thoughts and feelings)  - Guiding the caregiver in assessing the workability of these efforts  ***Learning materials***: My coping strategies worksheet and metaphors  ***Homework assignment***: Creative hopelessness worksheet and guided online ACT exercises |
| Week 3. ACT (Control vs. Willingness) | - Helping the caregiver acknowledge and consider experiencing the uncomfortable and unwanted emotions and thoughts as they are as an alternative to control  - Guiding the caregiver in exploring barriers to willingness and practicing exercises to facilitate overcoming barriers  ***Learning materials***: Metaphors, contacting my painful emotions worksheet  ***Homework assignment:*** Control as the problem worksheet, willingness worksheet, and guided online ACT exercises |
| Week 4. ACT (Acceptance) | - Fostering the caregiver’s willingness/acceptance using the case scenario and analysis exercise  - Coaching the caregiver in learning and applying strategies adapted to the caregiver’s patterns of experiential avoidance  ***Learning materials***: Case scenario exercise and coping with grief worksheet  ***Homework assignment:*** Guided online ACT exercises |
| Week 5. ACT (Cognitive defusion, observing self, and being present) | - Coaching the caregiver in: learning and applying exercises to step back or detach from unhelpful thoughts and emotions; practicing exercises to observe thoughts and emotions without judgement; and learning and practicing mindfulness exercises to promote maintaining contact with the present moment and living in the “here and now”  ***Learning materials***: Cognitive defusion exercises (e.g., passengers on the bus metaphor), exercises to observe thoughts and emotions (e.g., the continuous you/observer exercise), and mindfulness exercises (e.g., mindful breathing and body scan)  ***Homework assignment:*** Self-as-context worksheet and guided online ACT exercises |
| Week 6. ACT (Values and committed action) + Behavioral activation (BA) | - Assisting the caregiver with clarifying the core values that give them meaning/purpose and identifying potential barriers to living a values-focused life; identifying ways to overcome these barriers; setting a committed action plan; and using BA techniques (activity scheduling and monitoring) for involvement during week 6 in committed actions aligned with the caregiver’s personal values  ***Learning materials***: Exercises to help clarify values (e.g., tombstone exercise), demons on the boat metaphor, reflecting on your values, and my action plan worksheets  ***Homework assignment:*** Values worksheet and activity scheduling and monitoring worksheet |
| Week 7. ACT (Values and committed action) + BA | - Reviewing the caregiver’s performance of committed actions and mood in the past week and discussing barriers that hindered their committed actions during this period and ways to overcome those barriers and assisting the caregiver in revising their committed action plan for values-based living  - Coaching the caregiver in the use of mindfulness and defusion skills to follow through on the committed actions in the face of negative/painful thoughts and feelings  ***Learning materials***: My action plan worksheet  ***Homework assignment***: Activity scheduling and monitoring worksheet & guided online ACT exercises |
| Week 8. ACT + BA and Closure | - Same as the week 7 plus wrap up  ***Learning materials***: Joe the problem metaphor and my action plan worksheet  *Selected ACT online resources were provided after the completion of session 8 |

**Table S2.** Characteristics of Participants (N = 19)

| Variables | All (*n* = 19) | ACT group (*n* = 9) | Control group (*n* = 10) | Difference between groups |
| --- | --- | --- | --- | --- |
|  | *n* (%) or  M ± SD (range) | *n* (%) or  M ± SD (range) | *n* (%) or  M ± SD (range) | *p*- value |
| Age | 54.6 ± 15.1 (18–78) | 56 ± 20.1 (18–78) | 53.2 ± 10.7 (35–65) | .71 |
|  |  |  |  |  |
| Gender |  |  |  | N/A |
| Female | 19 (100%) | 9 (100%) | 10 (100%) |  |
|  |  |  |  |  |
| Race/Ethnicity |  |  |  | .56 |
| Black or African American | 9 (47.4%) | 5 (55.6%) | 4 (40%) |  |
| Non-Hispanic White | 8 (42.1%) | 4 (44.4%) | 4 (40%) |  |
| Hispanic or Latino | 1 (5.3%) |  | 1 (10%) |  |
| Native American or American Indian | 1 (5.3%) |  | 1 (10%) |  |
|  |  |  |  |  |
| Level of education |  |  |  | .86 |
| High school graduate | 2 (10.5%) | 1 (11.1%) | 1 (10%) |  |
| Technical, trade, or vocational school | 1 (5.3%) |  | 1 (10%) |  |
| Some college | 5 (26.3%) | 2 (22.2%) | 3 (30%) |  |
| Bachelor’s degree completed | 4 (21.1%) | 2 (22.2%) | 2 (20%) |  |
| Postgraduate | 7 (36.8%) | 4 (44.4%) | 3 (30%) |  |
|  |  |  |  |  |
| Marital status |  |  |  | .55 |
| Married/Living with a partner | 8 (42.1%) | 3 (33.3%) | 5 (50%) |  |
| Single/Never married | 6 (31.6%) | 4 (44.4%) | 2 (20%) |  |
| Divorced/Separated | 4 (21.1%) | 2 (22.2%) | 2 (20%) |  |
| Widowed | 1 (5.3%) |  | 1 (10%) |  |
|  |  |  |  |  |
| Employment status |  |  |  | .66 |
| Retired | 8 (42.1%) | 4 (44.4%) | 4 (40%) |  |
| Employed full time | 6 (31.6%) | 2 (22.2%) | 4 (40%) |  |
| Unemployed | 4 (21.1%) | 2 (22.2%) | 2 (20%) |  |
| Employed part time | 1 (5.3%) | 1 (11.1%) |  |  |
|  |  |  |  |  |
| Religion |  |  |  | .16 |
| Christian | 14 (73.7%) | 8 (88.9%) | 6 (60%) |  |
| Catholic | 2 (10.5%) |  | 2 (20%) |  |
| No religion | 2 (10.5%) |  | 2 (20%) |  |
| Jewish | 1 (5.3%) | 1 (11.1%) |  |  |
|  |  |  |  |  |
| Relationship to the relative with dementia |  |  |  | .28 |
| Daughter | 15 (78.9%) | 6 (66.7%) | 9 (90%) |  |
| Wife | 2 (10.5%) | 1 (11.1%) | 1 (10%) |  |
| Sister | 1 (5.3%) | 1 (11.1%) |  |  |
| Granddaughter | 1 (5.3%) | 1 (11.1%) |  |  |
|  |  |  |  |  |
| Living with the relative with dementia |  |  |  | 1.00 |
| Living together | 13 (68.4%) | 6 (66.7%) | 7 (70%) |  |
| Not living together | 6 (31.6%) | 3 (33.3%) | 3 (30%) |  |
|  |  |  |  |  |
| Caregiving hours per week |  |  |  | .74 |
| 40 hours or more | 9 (47.4%) | 4 (44.4%) | 5 (50%) |  |
| 21–39 hours | 4 (21.0%) | 2 (22.2%) | 2 (20%) |  |
| 9–20 hours | 5 (26.3%) | 3 (33.3%) | 2 (20%) |  |
| 8 hours or less | 1 (5.3%) |  | 1 (10%) |  |
|  |  |  |  |  |
| Other family members helping with the care |  |  |  | 1.00 |
| Yes | 11 (57.9%) | 5 (55.6%) | 6 (60%) |  |
| No | 8 (42.1%) | 4 (44.4%) | 4 (40%) |  |
|  |  |  |  |  |
| Participation in support group |  |  |  | .93 |
| Never attended | 10 (52.6%) | 5 (55.6%) | 5 (50%) |  |
| Currently attending (1 or 2 times per month) | 5 (26.3%) | 2 (22.2%) | 3 (30%) |  |
| Attended before, but not going anymore | 4 (21.1%) | 2 (22.2%) | 2 (20%) |  |
|  |  |  |  |  |
| Years since the relative’s diagnosis of dementia | 4.0 ± 2.6 (1–10) | 3.6 ± 2.7 (1–10) | 4.4 ± 2.5 (1–9) | .52 |
|  |  |  |  |  |
| Years of caregiving | 3.9 ± 2.4 (1–10) | 2.9 ± 1.4 (1–5.7) | 4.7 ± 2.8 (1.25–10) | .11 |
|  |  |  |  |  |
| Type of dementia in the relative with dementia |  |  |  | .73 |
| Alzheimer’s disease | 10 (52.6%) | 6 (66.7%) | 4 (40%) |  |
| Not sure | 4 (21.1%) | 1 (11.1%) | 3 (30%) |  |
| Dementia with Lewy bodies | 3 (15.8%) | 1 (11.1%) | 2 (20%) |  |
| Vascular dementia | 2 (10.5%) | 1 (11.1%) | 1 (10%) |  |
|  |  |  |  |  |
| Stage of dementia in the relative with dementia |  |  |  | .78 |
| Early/Mild | 2 (10.5%) | 1 (11.1%) | 1 (10%) |  |
| Middle/Moderate | 11 (57.9%) | 5 (55.6%) | 6 (60%) |  |
| Late/Severe | 4 (21.1%) | 2 (22.2%) | 2 (20%) |  |
| Not sure | 2 (10.5%) | 1 (11.1%) | 1 (10%) |  |
|  |  |  |  |  |
| BPSD of the relative with dementia  (Participants checked all that apply) |  |  |  | N/A |
| Anxiety and agitation | 16 (84.2%) | 8 (88.9%) | 8 (80%) |  |
| Repetition | 14 (73.7%) | 8 (88.9%) | 6 (60%) |  |
| Depression | 9 (47.4%) | 3 (33.3%) | 6 (60%) |  |
| Aggression and anger | 9 (47.4%) | 6 (66.7%) | 3 (30%) |  |
| Hallucinations | 8 (42.1%) | 4 (44.4%) | 4 (40%) |  |
| Resistance to and refusal of care | 8 (42.1%) | 3 (33.3%) | 5 (50%) |  |
| Sleep issues | 7 (36.8%) | 3 (33.3%) | 4 (40%) |  |
| Suspicions and delusions | 7 (36.8%) | 4 (44.4%) | 3 (30%) |  |
| Wandering | 3 (15.8%) | 2 (22.2%) | 1 (10%) |  |
|  |  |  |  |  |

Abbreviations: ACT, acceptance and commitment therapy; BPSD, behavioral and psychological symptoms of dementia; M, mean; N/A, not applicable; SD, standard deviation.

*Statistically significant at *p* < 0.05.

**Table S3.** Between-Group Differences in Depended Variables at Pretest

| Independent Samples t Test | | | | | | | | | | | |
| --- | --- | --- | --- | --- | --- | --- | --- | --- | --- | --- | --- |
| Variables | | Levene’s Test for Equality of Variances | | t-test for Equality of Means | | | | | | | |
|  |  | F | Sig. | t | df | Significance | | Mean Difference | Std. Error Difference | 95% Confidence Interval of the Difference | |
|  |  |  |  |  |  | One-Sided *p* | Two-Sided *p* |  |  | Lower | Upper |
| DASS-21 Depression | Equal variances assumed | 3.572 | 0.076 | -1.090 | 17 | 0.146 | 0.291 | -2.600 | 2.386 | -7.634 | 2.434 |
| DASS-21 Anxiety | Equal variances assumed | 0.109 | 0.745 | -1.120 | 17 | 0.139 | 0.278 | -2.100 | 1.874 | -6.055 | 1.855 |
| DASS-21  Stress | Equal variances assumed | 2.678 | 0.120 | 0.055 | 17 | 0.478 | 0.957 | 0.100 | 1.821 | -3.741 | 3.941 |
| ZBI | Equal variances assumed | 2.914 | 0.106 | -0.571 | 17 | 0.288 | 0.576 | -2.089 | 3.660 | -9.810 | 5.632 |
| MM-CGI-BF | Equal variances assumed | 0.035 | 0.855 | -1.597 | 17 | 0.064 | 0.129 | -3.511 | 2.199 | -8.150 | 1.128 |
| CGQ | Equal variances assumed | 0.919 | 0.351 | -1.154 | 17 | 0.132 | 0.264 | -7.422 | 6.430 | -20.988 | 6.143 |
| WHOQOL‑BREF  - Psychological | Equal variances assumed | 1.420 | 0.250 | 0.248 | 17 | 0.404 | 0.807 | 0.060 | 0.241 | -0.448 | 0.567 |
| AAQ-II | Equal variances assumed | 0.156 | 0.698 | -0.623 | 17 | 0.271 | 0.542 | -3.222 | 5.174 | -14.138 | 7.694 |
| CFQ-7 | Equal variances assumed | 0.094 | 0.762 | -0.819 | 17 | 0.212 | 0.424 | -3.911 | 4.778 | -13.992 | 6.170 |
| EMAS | Equal variances assumed | 0.034 | 0.855 | -1.051 | 17 | 0.154 | 0.308 | -3.700 | 3.521 | -11.128 | 3.728 |
| SCS-SF | Equal variances assumed | 1.423 | 0.249 | 0.884 | 17 | 0.195 | 0.389 | 0.153 | 0.173 | -0.212 | 0.517 |

Abbreviations: AAQ-II, Acceptance and Action Questionnaire-II; CGQ, Caregiver Guilt Questionnaire; CFQ-7, Cognitive Fusion Questionnaire-7; DASS-21, Depression, Anxiety and Stress Scale-21; EMAS, Engagement in Meaningful Activities Survey; MM-CGI-BF, Marwit–Meuser Caregiver Grief Inventory-Brief-Form; SCS-SF, Self-Compassion Scale-Short Form; WHOQOL‑BREF, World Health Organization Quality of Life Assessment‑BREF; ZBI, Zarit Burden Interview.

*Statistically significant at *p* < 0.05.

**Table S4.** Within-Group Comparisons in Dependent Variables

| Variables | ACT group (*n* = 9) | | | | | Control group (*n* = 10) | | | | |
| --- | --- | --- | --- | --- | --- | --- | --- | --- | --- | --- |
|  | Pretest  M ± SD (Median, IQR) | Posttest  M ± SD (Median, IQR) | 1-mo F/U  M ± SD (Median, IQR) | *p* (effect size)  pretest-posttest | *p* (effect size)  pretest-F/U | Pretest  M ± SD (Median, IQR) | Posttest  M ± SD (Median, IQR) | 1-mo F/U  M ± SD (Median, IQR) | *p* (effect size)  pretest-posttest | *p* (effect size)  pretest-F/U |
| DASS-21 |  |  |  |  |  |  |  |  |  |  |
| Depression (-) | 8 ± 6.93  (6, 11) | 5.22 ± 2.73  (5, 4.5) | 6.33 ± 4.9  (6, 3) | .260  (.27) | .767  (.07) | 5.4 ± 2.88  (5.5, 5.5) | 4.9 ± 2.56 (4.5, 5.25) | 4.2 ± 3.88  (3.5, 6.25) | .857  (.04) | .478  (.16) |
| Anxiety (-) | 6 ± 4.24  (6, 4.5) | 3.89 ± 1.62  (4, 3.5) | 5.56 ± 6.06  (3, 8.5) | .182  (.31) | .812  (.06) | 3.9 ± 3.93  (3, 8.25) | 3 ± 3.06  (2, 4) | 3 ± 3.02  (2, 3.25) | .263  (.25) | .251  (.26) |
| Stress (-) | 10 ± 2.6  (9, 4) | 6.22 ± 2.82  (7, 3) | 7.33 ± 4.15  (7, 4.5) | .011*  (.60) | .138  (.35) | 10.10 ± 4.86  (9, 7.75) | 6.7 ± 4.06  (6, 4.75) | 6.4 ± 4.97  (6, 9.5) | .029*  (.49) | .050*  (.44) |
|  |  |  |  |  |  |  |  |  |  |  |
| ZBI (-) | 28.89 ± 6.21  (29, 6) | 24.89 ± 7.22  (26, 14) | 23.5 ± 7.5  (25, 13.25) | .233  (.28) | .110  (.38) | 26.8 ± 9.25  (29.5, 17) | 23.3 ± 8.33  (25, 15.75) | 20.6 ± 11.54  (19.5, 21.5) | .065  (.41) | .096  (.37) |
|  |  |  |  |  |  |  |  |  |  |  |
| MM-CGI-BF (-) | 21.11 ± 4.7  (23, 7.5) | 18.22 ± 4.35  (18, 5.5) | 18.22 ± 5.43  (20, 6.5) | .049*  (.46) | .049*  (.46) | 17.6 ± 4.86  (18.5, 7) | 19 ± 5.79  (19, 8.5) | 16.7 ± 6.4  (18, 9.5) | .398  (.19) | .483  (.16) |
|  |  |  |  |  |  |  |  |  |  |  |
| CGQ (-) | 44.22 ± 15.29  (47, 31.5) | 33.22 ± 17.44  (32, 32) | 34 ± 18.1  (35, 28.5) | .044*  (.48) | .236  (.28) | 36.8 ± 12.73  (39, 20.5) | 27.2 ± 15.62  (22, 27.25) | 28.4 ± 18.12  (24, 29) | .012*  (.56) | .030*  (.49) |
|  |  |  |  |  |  |  |  |  |  |  |
| WHOQOL‑BREF  - Psychological | 18.11 ± 5.06  (19, 7.5) | 21.11 ± 4.23  (22, 5) | 21.67 ± 4.09  (23, 6) | .05*  (.46) | .035*  (.50) | 18.80 ± 3.65  (18.5, 7.25) | 19.60 ± 2.95  (20.0, 5.5) | 21.20 ± 4.44  (20.5, 8.0) | .610  (.11) | .064  (.41) |
|  |  |  |  |  |  |  |  |  |  |  |
| AAQ-II (-) | 24.22 ± 10.58  (25, 15.5) | 19.56 ± 8.03  (19, 15) | 16.56 ± 6.91  (17, 9) | .175  (.32) | .034*  (.50) | 21 ± 11.83  (19.5, 14.25) | 19.5 ± 11.47  (18, 14.5) | 20.9 ± 11.47  (22.5, 14.5) | .552  (.13) | .673  (.09) |
|  |  |  |  |  |  |  |  |  |  |  |
| CFQ-7 (-) | 26.11 ± 11.04  (27, 16.5) | 23 ± 10.06  (21, 18.5) | 22.44 ± 10.26  (28, 19) | .515  (.15) | .513  (.15) | 22.2 ± 9.8  (21, 16) | 22.3 ± 11.05  (21, 19.5) | 20.6 ± 11.28  (18.5, 17.25) | 1.000  (.00) | .088  (.38) |
|  |  |  |  |  |  |  |  |  |  |  |
| EMAS | 32 ± 7.65  (32, 12.5) | 37.33 ± 5.48  (36, 9) | 38.44 ± 6.31  (39, 12.5) | .066  (.43) | .066  (.43) | 28.3 ± 7.67  (28, 8.75) | 28.4 ± 9  (27, 13.5) | 31.3 ± 9.07  (30.5, 15) | .888  (.03) | .063  (.42) |
|  |  |  |  |  |  |  |  |  |  |  |
| SCS-SF | 2.95 ± .75  (2.92, .84) | 3.25 ± .50  (3.08, .71) | 3.44 ± .63  (3.5, .83) | .075  (.42) | .049*  (.46) | 3.01 ± .58  (3.13, .68) | 3.44 ± .71  (3.33, 1.23) | 3.43 ± .91  (3.33, 1.23) | .011*  (.57) | .038*  (.46) |
|  |  |  |  |  |  |  |  |  |  |  |

*Note.* A minus sign in parentheses indicates that a decline in each variable means positive outcomes.

Abbreviations: AAQ-II, Acceptance and Action Questionnaire-II; CGQ, Caregiver Guilt Questionnaire; CFQ-7, Cognitive Fusion Questionnaire-7; DASS-21, Depression, Anxiety and Stress Scale-21; EMAS, Engagement in Meaningful Activities Survey; F/U, follow-up; IQR, interquartile range; M, mean; MM-CGI-BF, Marwit–Meuser Caregiver Grief Inventory-Brief-Form; SCS-SF, Self-Compassion Scale-Short Form; SD, standard deviation; WHOQOL‑BREF, World Health Organization Quality of Life Assessment‑BREF; ZBI, Zarit Burden Interview.

*Statistically significant at *p* ≤ 0.05.

**Table S5.** Between-Group Comparisons in Dependent Variables

| Variables | ACT group (*n* = 9) | | Control group (*n* = 10) | | Between-group comparison  Posttest-pretest | | | Between-group comparison  1-month F/U-pretest | | |
| --- | --- | --- | --- | --- | --- | --- | --- | --- | --- | --- |
|  | Posttest-pretest  M ± SD  (Median, IQR) | 1-month F/U-pretest  M ± SD  (Median, IQR) | Posttest-pretest  M ± SD  (Median, IQR) | 1-month F/U-pretest  M ± SD  (Median, IQR) | Z | *p* | Effect size (r) | Z | *p* | Effect size (r) |
| DASS-21 |  |  |  |  |  |  |  |  |  |  |
| Depression (-) | -2.78 ± 5.89  (-1, 9.5) | -1.67 ± 8.89  (1, 13.5) | -.5 ± 2.92  (-.5, 3.5) | -1.2 ± 4.76  (-.5, 7.25) | -.66 | .549 | .15 | -.25 | .842 | .06 |
| Anxiety (-) | -2.11 ± 4.31  (-2, 5.5) | -.44 ± 7.55  (-1, 12) | -.9 ± 2.23  (0, 3.25) | -.9 ± 2.56  (-1, 1.5) | -.99 | .356 | .23 | -.04 | .968 | .01 |
| Stress (-) | -3.78 ± 2.82  (-3, 4.5) | -2.67 ± 5.2  (-3, 9) | -3.4 ± 4.55  (-3, 4.75) | -3.7 ± 5.77  (-2.5, 5.75) | -.58 | .604 | .13 | -.12 | .905 | .03 |
|  |  |  |  |  |  |  |  |  |  |  |
| ZBI (-) | -4 ± 8.11  (-2, 13) | -5.39 ± 8.42  (-6, 16.25) | -3.5 ± 5.4  (-4, 10) | -6.2 ± 11.83  (-2.5, 11.75) | -.16 | .905 | .04 | .00 | 1.000 | .00 |
|  |  |  |  |  |  |  |  |  |  |  |
| MM-CGI-BF (-) | -2.89 ± 3.52  (-3, 3.5) | -3.33 ± 3.35  (-3, 6) | 1.4 ± 4.03  (1.5, 5.25) | -.9 ± 4.89  (0, 9.5) | -2.14 | .035* | .49 | -1.23 | .243 | .28 |
|  |  |  |  |  |  |  |  |  |  |  |
| CGQ (-) | -11 ± 13.22  (-7, 23.5) | -10.22 ± 18.08  (-5, 35.5) | -9.6 ± 11  (-6.5, 14.5) | -8.4 ± 11.11  (-6, 15.5) | -.16 | .905 | .04 | -.25 | .842 | .06 |
|  |  |  |  |  |  |  |  |  |  |  |
| WHOQOL‑BREF  - Psychological | 3.00 ± 3.61  (4, 1) | 3.56 ± 3.97  (4, 2) | .80 ± 3.05  (0, 3.25) | 2.40 ± 3.86  (2, 4.25) | -1.40 | .182 | .32 | -.74 | .497 | .17 |
|  |  |  |  |  |  |  |  |  |  |  |
| AAQ-II (-) | -4.67 ± 8.8  (0, 11) | -7.67 ± 8.93  (-7, 14.5) | -1.5 ± 6.6  (-.5, 8.25) | -.1 ± 3.81  (0, 5) | -.74 | .497 | .17 | -1.93 | .053 | .44 |
|  |  |  |  |  |  |  |  |  |  |  |
| CFQ-7 (-) | -3.11 ± 9.74  (-2, 12) | -3.67 ± 11.87  (-1, 16) | .1 ± 4.38  (0, 7) | -1.6 ± 3.06  (-1, 3.75) | -.49 | .661 | .11 | -.25 | .842 | .06 |
|  |  |  |  |  |  |  |  |  |  |  |
| EMAS | 5.33 ± 7.68  (4, 11.5) | 6.44 ± 9.32  (5, 13.5) | .1 ± 5.69  (0, 8.5) | 3 ± 5.08  (2, 6.5) | -1.43 | .156 | .33 | -.94 | .356 | .22 |
|  |  |  |  |  |  |  |  |  |  |  |
| SCS-SF | .30 ± .49  (.17, .63) | .48 ± .56  (.58, 1.13) | .43 ± .38  (.34, .79) | .42 ± .53  (.38, .92) | -.62 | .549 | .14 | -.04 | .97 | .00 |
|  |  |  |  |  |  |  |  |  |  |  |

*Note.* A minus sign in parentheses indicates that a decline in each variable means positive outcomes.

Abbreviations: AAQ-II, Acceptance and Action Questionnaire-II; CGQ, Caregiver Guilt Questionnaire; CFQ-7, Cognitive Fusion Questionnaire-7; DASS-21, Depression, Anxiety and Stress Scale-21; EMAS, Engagement in Meaningful Activities Survey; F/U, follow-up; IQR, interquartile range; M, mean; MM-CGI-BF, Marwit–Meuser Caregiver Grief Inventory-Brief-Form; SCS-SF, Self-Compassion Scale-Short Form; SD, standard deviation; WHOQOL‑BREF, World Health Organization Quality of Life Assessment‑BREF; ZBI, Zarit Burden Interview.

*Statistically significant at *p* < 0.05.
